# Identification of a putative novel polycyclic aromatic hydrocarbon-biodegrading gene cluster in a marine *Roseobacteraceae* bacterium *Sagittula* sp. MA-2

**DOI:** 10.1128/spectrum.01074-24

**Published:** 2024-11-27

**Authors:** Mayuko Abe, Miharu Sakai, Robert A. Kanaly, Jiro F. Mori

**Affiliations:** 1 Graduate School of Nanobioscience, Yokohama City University, Yokohama, Japan; Swansea University, Swansea, United Kingdom

**Keywords:** *Roseobacteraceae*, *Sagittula*, polycyclic aromatic hydrocabons, biodegradation, comparative genomics

## Abstract

The ability to biodegrade polycyclic aromatic hydrocarbons (PAHs) and the catabolic enzymes responsible for PAH biotransformation in marine bacteria belonging to the family *Roseobacteraceae* remain largely unexplored despite their wide distribution and highly diverse physiological traits. A bacterial isolate within *Roseobacteraceae* originating from coastal seawater, *Sagittula* sp. strain MA-2, that biotransformed phenanthrene and utilized it as a growth substrate was found to possess a putative PAH-degrading gene cluster on one of the eight circular plasmids in its genome. Subsequent comprehensive investigations utilizing bacterial genomes in public databases revealed that gene clusters potentially homologous to this newly found cluster are widely but heterogeneously distributed within *Roseobacteraceae* and a few non-*Roseobacteraceae* (*Paracoccaceae* and *Rhizobiaceae*) strains from saline environments. Catabolic functions of the enzymes encoded in strain MA-2 were predicted through the profiling of phenanthrene biotransformation products by liquid chromatography-electrospray ionization high-resolution mass spectrometry and substrate docking simulations using predicted three-dimensional structures of selected proteins, and phenanthrene biodegradation pathways were proposed. Strain MA-2 appeared to biodegrade phenanthrene via two separated, concurrent pathways, namely the salicylate and phthalate pathways. This study serves as the first investigation into the functional genes potentially responsible for PAH biodegradation conserved in *Roseobacteraceae* bacteria, expanding scientific understanding of the physiological repertoire evolved in this ubiquitous marine bacterial group.

**IMPORTANCE:**

The ocean is often characterized as the terminal destination for persistent polycyclic aromatic hydrocarbon (PAH) environmental pollutants; however, the ability to biodegrade PAHs and the corresponding enzymes conserved among marine bacteria are less understood compared to their terrestrial counterparts. A marine bacterial isolate, *Sagittula* sp. strain MA-2, belonging to the family *Roseobacteraceae*—a widely distributed and physiologically diverse marine bacterial group—was found to possess a functional gene cluster encoding enzymes potentially responsible for PAH biodegradation in its genome and exhibit the ability to biodegrade the three-ring PAH, phenanthrene. Intriguingly, gene clusters potentially homologous to this cluster were also distributed broadly across genomes from different *Roseobacteraceae* genera in public databases, which has not been previously investigated. The knowledge provided here expands our understanding of the physiology of *Roseobacteraceae* and may be applied to explore biotechnologically useful bacteria that contribute to the remediation of polluted marine environments or high-salinity wastewater.

## INTRODUCTION

Bacterial biodegradation of polycyclic aromatic hydrocarbons (PAHs), which are ubiquitous and persistent environmental pollutants that possess acute toxicity and genotoxicity, is driven through multiple steps of biotransformation catalyzed by specialized enzymes that have evolved and are conserved in certain bacterial groups. These enzymes are represented by aromatic ring-hydroxylating dioxygenases (ARHDs) that catalyze the initial biotransformation processes by adding two oxygen atoms to the aromatic rings (1, 2), and aromatic ring-cleaving dioxygenases (ARCDs) that cleave the oxidized aromatic rings (3). Through genomic investigations of environmental bacteria with PAH-degrading abilities, functional genes encoding these cooperative enzymes have been identified to be conserved as gene clusters in their genomes (4). Past studies investigating the bacterial PAH-degrading gene clusters have largely focused on bacteria in terrestrial environments; the *nah*/*pah* genes in *Pseudomonas* spp. (5, 6), the *ahd*/*bph*/*phn* genes in sphingomonads (7–9), and the *nid* genes in *Mycobacterium* spp. (10, 11) have been found as conserved in each bacterial group and extensively characterized. These known functional genes have been utilized as biomarkers to explore and identify bacterial players with PAH biodegradation abilities within bacterial communities (12, 13).

In marine environments, in contrast to terrestrial environments, bacterial PAH biodegradation pathways and enzymes involved are not well understood. Despite this, the ocean is frequently characterized as the terminal destination for PAH pollutants and marine microorganisms are considered to play crucial roles in the elimination of these pollutants (14, 15). The known enzymes responsible for the biodegradation of PAHs in marine bacteria include the Nah enzymes found in *Stutzerimonas stutzeri* (formerly *Pseudomonas stutzeri*; family *Pseudomonadaceae*) and the Phn enzymes from *Cycloclasticus* spp. (family *Piscirickettsiaceae*); similar to bacteria from terrestrial habitats, genes encoding these enzymes were identified forming gene clusters in their genomes (16, 17). Meanwhile, in the family *Roseobacteraceae*, one of the most commonly distributed bacterial groups in marine environments comprising over 120 genera with highly diverse physiological traits (18, 19), few cases of investigated PAH biodegradation abilities and their enzymes involved have been reported. Past studies have revealed the possibility that various bacterial genera within *Roseobacteraceae* contribute to PAH biodegradation through co-metabolism or their activities as consortia (20–22); however, their abilities to utilize PAHs as growth substrates have rarely been found. A bacterial strain belonging to *Roseobacteraceae*, *Celeribacter indicus* strain P73^T^, isolated from deep-sea sediment, was characterized as capable of growing on fluoranthene, and its enzymes responsible for fluoranthene biodegradation were proposed (23). It represents the only reported case of a genomic investigation of *Roseobacteraceae* bacteria that biodegrade PAHs and grow on them as the sole carbon source, to the best of our knowledge.

In the current study, a marine bacterium belonging to *Roseobacteraceae*, *Sagittula* sp. strain MA-2 (JCM 36213), isolated from coastal seawater and whose complete genome sequence was recently announced (24), was found to be capable of growing on phenanthrene as the sole carbon source. Based on the results of genomic analyses combined with the profiling of phenanthrene biotransformation products, functional genes putatively involved in PAH biodegradation in strain MA-2 were identified. Through further comprehensive investigations of bacterial genomes in public databases using the genes identified in strain MA-2 as a model, this study aimed to clarify the conservation and diversity of potential PAH-degrading genes within *Roseobacteraceae*, which has received limited investigation to date.

## RESULTS

### Growth of strain MA-2 on phenanthrene as the sole carbon source

**Fig 1:**
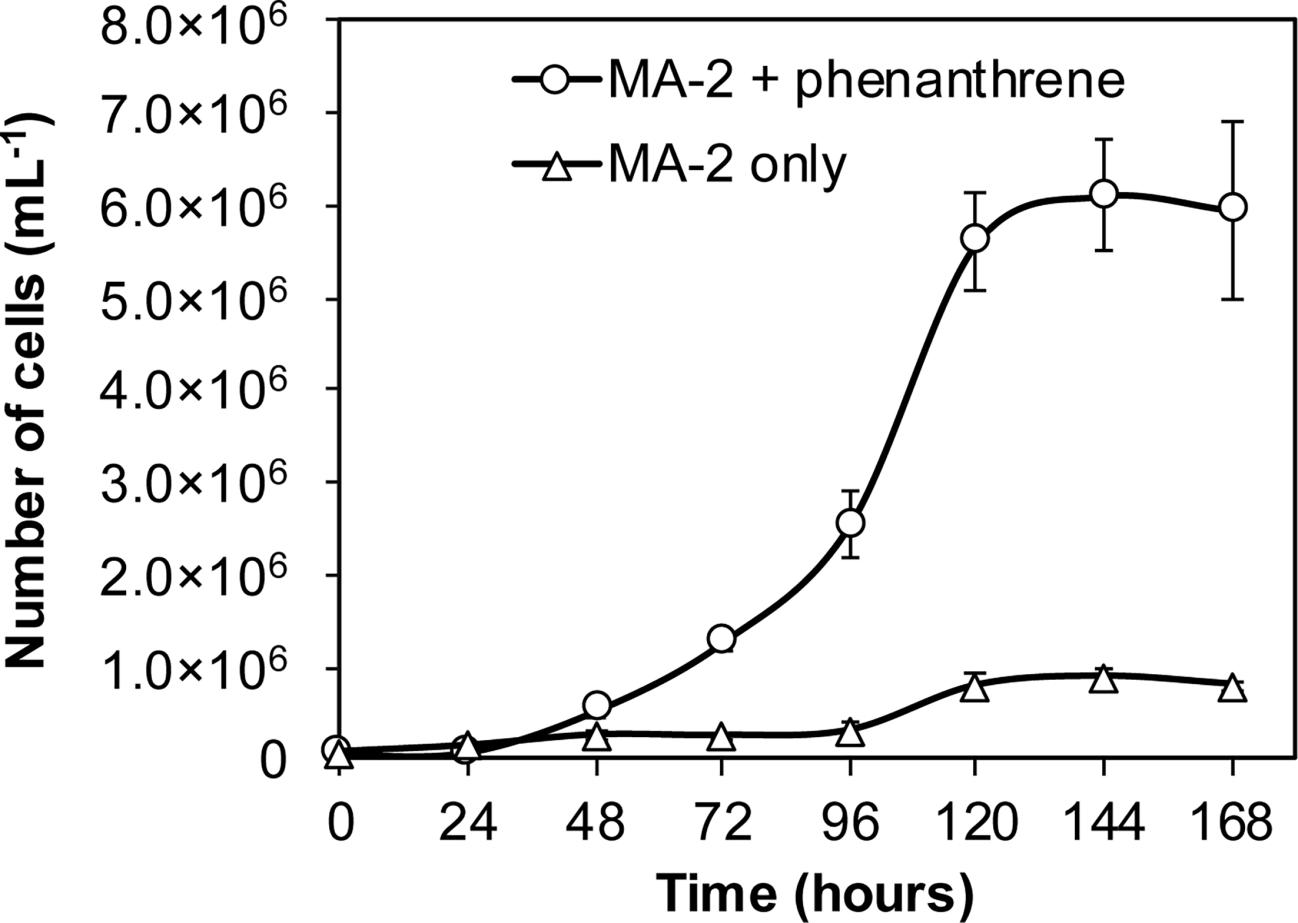
Cell numbers in strain MA-2 cultures with 50 mg L^−1^ phenanthrene as the sole carbon source (circle; *n* = 3) or without carbon source (triangle; *n* = 3).

**Fig 2:**
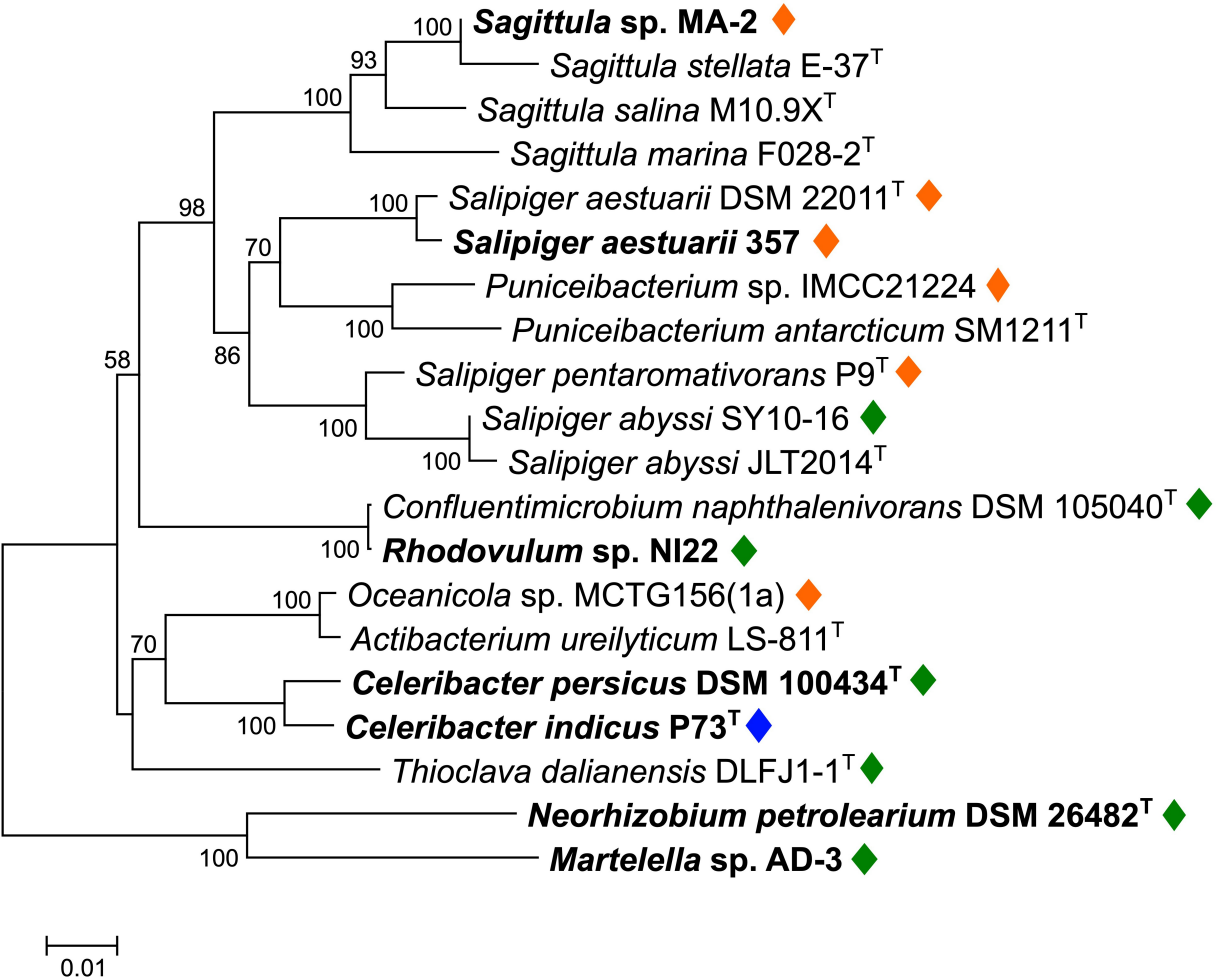
Neighbor-joining phylogenetic tree of 16S rRNA genes of strain MA-2 and selected reference bacterial strains belonging to the families *Roseobacteraceae* (16 strains), *Paracoccaceae* (*Thioclava dalianensis* DLFJ1-1^T^), or *Rhizobiaceae* (two strains; *Neorhizobium petrolearium* DSM 26482^T^ and *Martelella* sp. AD-3). Strains previously reported to have the ability to grow on PAHs as the sole carbon source are highlighted in boldface. Diamond symbols indicate strains identified to possess the PAH-degrading gene cluster in their genomes (orange, type A; green, type B; blue, type C as shown in Fig. 3). The tree was created with 1,000 bootstrap iterations, and values below 50% are not displayed.

*Sagittula* sp. strain MA-2 was isolated from a marine bacterial consortium enriched with phenanthrene and was repeatedly sub-cultured in artificial sea water (ASW) medium with 50 mg L^−1^ gentisic acid as the sole carbon source (24). When strain MA-2 cells were inoculated into a new ASW medium containing 50 mg L^−1^ phenanthrene as the sole carbon source, an increase in cell numbers was observed. The initial cell count ranged from 50,000 to 70,000 cells mL^−1^ immediately after inoculation, reaching approximately 6.0 × 10^6^ cells mL^−1^ at the stationary phase after 120 hours of incubation (Fig. 1). Taxonomic analyses revealed that strain MA-2 exhibited 99.8% 16S rRNA gene sequence identity and 89.4% average nucleic acid identity (ANI) to *Sagittula stellata* E-37^T^, placing it within the genus *Sagittula* in the family *Roseobacteraceae* (Fig. 2).

### Identification of the putative PAH-degrading gene cluster in the strain MA-2 genome

**Fig 3:**
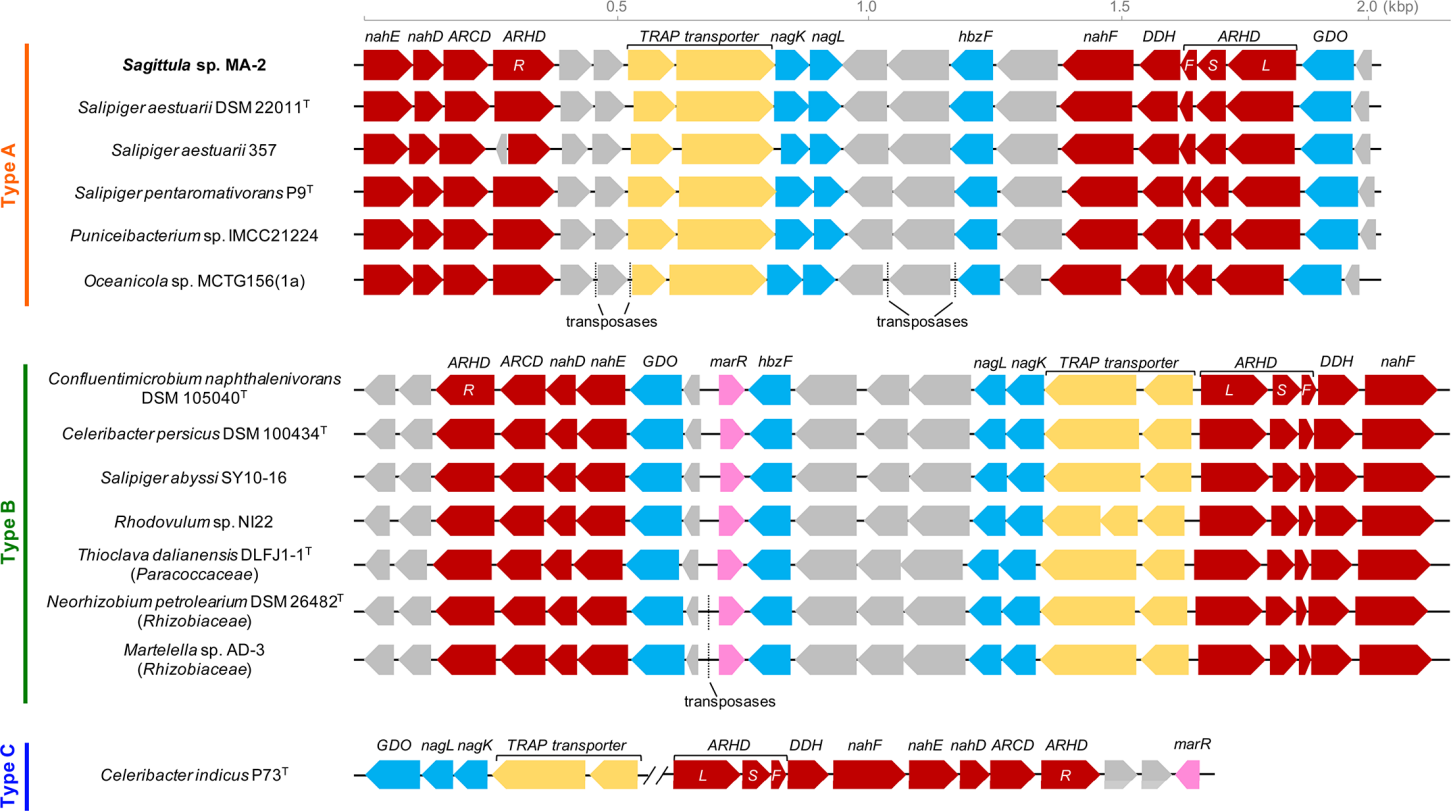
Putative PAH-degrading gene cluster shared in the genomes of *Roseobacteraceae* (10 strains), *Paracoccaceae* (*Thioclava dalianensis* DLFJ1-1^T^), and *Rhizobiaceae* (two strains; *Neorhizobium petrolearium* DSM 26432^T^ and *Martelella* sp. AD-3). The gene cluster was categorized into three sub-classes: types A, B, and C. ARHD, aromatic ring-hydroxylating dioxygenase (L, large subunit; S, small subunit; F, ferredoxin subunit; R, ferredoxin reductase subunit); DDH, dihydrodiol dehydrogenase; ARCD, aromatic ring-cleaving dioxygenase; and GDO, gentisate 1,2-dioxygenase.

The genome of strain MA-2 comprised a single circular chromosome with a size of 4.7 Mbp and eight circular plasmids ranging from 19.5 to 361 kbp in size (24). The candidate functional genes involved in aromatic hydrocarbon biodegradation were screened from the entire genome of strain MA-2 (summarized in Table S1). As functional marker genes for PAH biodegradation, genes encoding four subunits (large, small, ferredoxin, and ferredoxin reductase) of aromatic ring-hydroxylating dioxygenases were identified, exhibiting 47%–61% amino acid identities with subunits of biphenyl 2,3-dioxygenase (BphAa-d) of *Rhodococcus jostii* RHA1 (Table 1). *R. jostii* RHA1 was previously demonstrated to transform different PAHs including phenanthrene (25). These four genes were located within a gene cluster (IMG gene ID 8019787688–8019787708) found on one of the eight plasmids (plasmid p6; 162,034 bp; Fig. S1), and this gene cluster also included genes predicted to encode enzymes involved in aromatic hydrocarbon biodegradation, such as BphB-like dihydrodiol dehydrogenase (DDH), catechol 2,3-dioxygenase-like aromatic ring-cleaving dioxygenase, Nah enzymes (NahD, NahE, and NahF) for phenanthrene/naphthalene biodegradation, gentisate 1,2-dioxygenase (GDO), HbzF, and Nag enzymes (NagK and NagL) for gentisic acid biodegradation (Table 1; Fig. 3).

**TABLE 1:** List of functional genes included in the putative PAH-degrading gene cluster in the strain MA-2 genome

IMG gene ID	Predicted products	Related aromatic hydrocarbon-degrading enzymes (organism, UniProtKB ID, aa identity)
8019787688	NahE, *trans-o-*hydroxybenzylidenepyruvate hydratase-aldolase (331 aa)	NahE, *trans-o*-hydroxybenzylidenepyruvate hydratase-aldolase (*Pseudomonas putida* strains, P0A144, 58.8%)
8019787689	NahD, 2-hydroxychromene-2-carboxylate isomerase (199 aa)	NahD, 2-hydroxychromene-2-carboxylate isomerase (*Pseudomonas putida* strains, Q51948, 41.7%)
8019787690	Aromatic ring-cleaving dioxygenase (301 aa)	Catechol 2,3-dioxygenase (*Rhodococcus opacus* R7, 47.2%)
8019787691	Aromatic ring-hydroxylating dioxygenase ferredoxin reductase subunit (408 aa)	BphAd, biphenyl 2,3-dioxygenase ferredoxin reductase component (*Rhodococcus jostii* RHA1, Q0S032, 31.9%)
8019787692	Outer membrane protein (219 aa)	–^*a*^
8019787693	Glutathione *S*-transferase (202 aa)	–
8019787694	TRAP transporter TAXI family solute receptor (319 aa)	–
8019787695	TRAP transporter 4TM/12TM fusion protein (654 aa)	–
8019787696	NagK, fumarylpyruvate hydrolase (235 aa)	NagK, fumarylpyruvate hydrolase (*Ralstonia* sp. strains, O86042, 46.6%)
8019787697	NagL, maleypyruvate isomerase (215 aa)	NagL, maleypyruvate isomerase (*Ralstonia* sp. strains, O86043, 49.1%)
8019787698	Hydroxymethylglutaryl-CoA lyase (300 aa)	–
8019787699	Acyl-CoA transferase (408 aa)	–
8019787700	HbzF, maleylpyruvate hydrolase (285 aa)	HbzF, maleylpyruvate hydrolase (*Pseudomonas alcaligenes* strains, Q0QFQ3, 57.0%)
8019787701	Cytochrome P450 (412 aa)	–
8019787702	NahF, salicylaldehyde dehydrogenase (485 aa)	NahF, salicylaldehyde dehydrogenase (*Pseudomonas putida* strains, P0A391, 59.8%)
8019787703	Dihydrodiol dehydrogenase (DDH) (275 aa)	BphB, *cis*-2,3-dihydrobiphenyl-2,3-diol dehydrogenase (*Pseudomonas putida* strains, P72220, 54.0%)
8019787704	Aromatic ring-hydroxylating dioxygenase ferredoxin subunit (110 aa)	BphAc, biphenyl 2,3-dioxygenase ferredoxin component (*Rhodococcus jostii* RHA1, Q53124, 47.1%)
8019787705	Aromatic ring-hydroxylating dioxygenase small subunit (186 aa)	BphAb, biphenyl 2,3-dioxygenase subunit beta (*Rhodococcus jostii* RHA1, Q53123, 61.0%)
8019787706	Aromatic ring-hydroxylating dioxygenase) large subunit (451 aa)	BphAa, biphenyl 2,3-dioxygenase subunit alpha (*Rhodococcus jostii* RHA1, Q53122, 59.1%)
8019787707	Gentisate 1,2-dioxygenase (350 aa)	XlnE, gentisate 1,2-dioxygenase (*Pseudomonas alcaligenes* strains, Q9S3U6, 66.9%)
8019787708	2Fe-2S ferredoxin (109 aa)	–

^*a*^ –, not related to known aromatic hydrocarbon-degrading enzymes.

**Fig 4:**
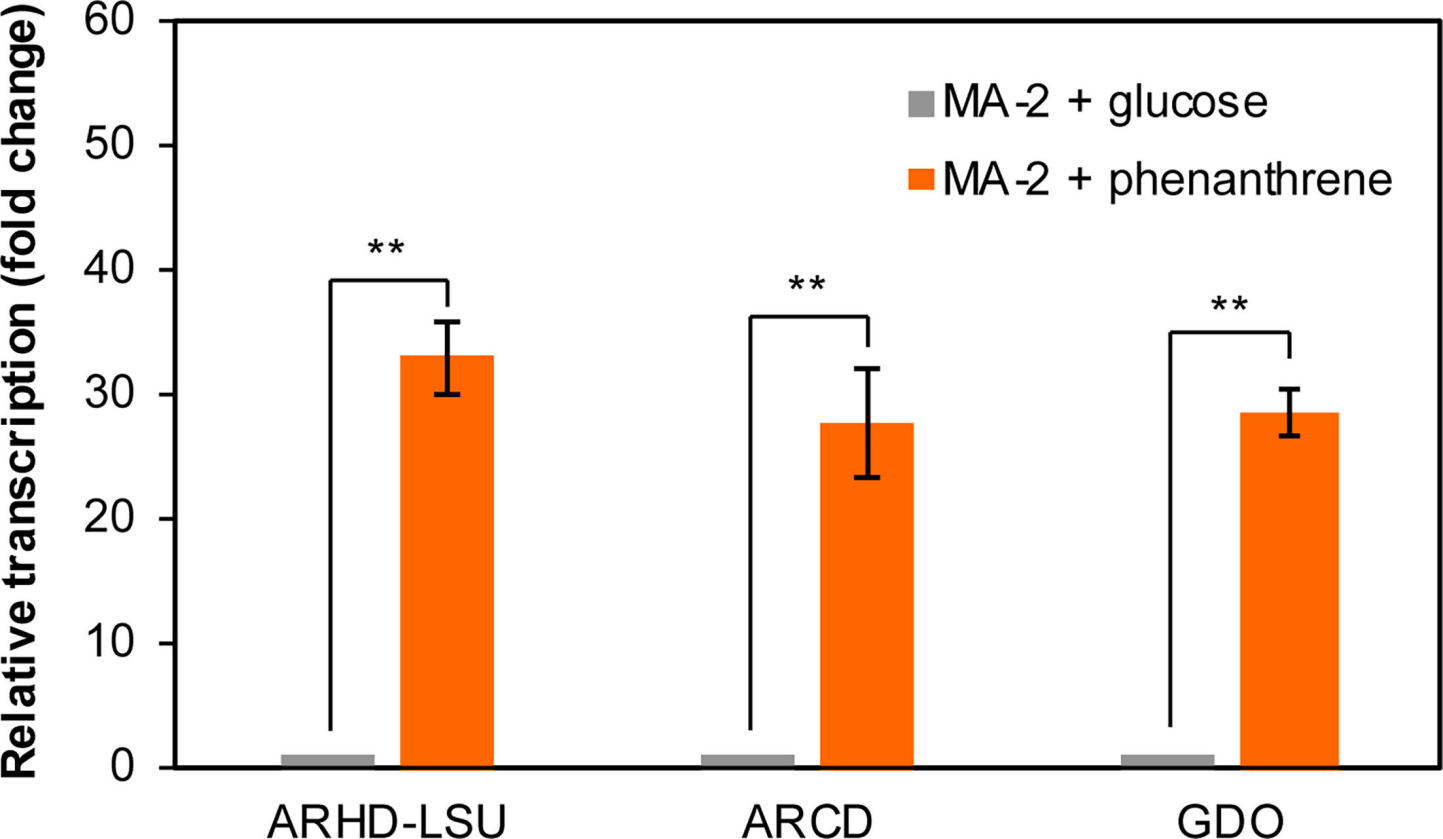
Relative transcriptional levels of genes encoding ARHD-LSU, ARCD, or GDO in strain MA-2 cells cultivated with phenanthrene (orange bars) compared to the cells cultivated with glucose (gray bars). Relative transcriptional levels were determined using the 2^-ΔΔCt^ method with the *repC* gene as the internal reference. **P < 0.01 (Welch’s *t*-test); error bars indicate standard deviations (*n* = 3).

Consistent with the observed amino acid sequence homology to known PAH-degrading genes, reverse transcription-quantitative PCR (RT-qPCR) results indicated that the expressions of genes encoding the large subunit of ARHD (ARHD-LSU), ARCD, and GDO in this gene cluster were significantly upregulated when strain MA-2 cells were cultured with 50 mg L^−1^ phenanthrene, showing 27.8- to 33.0-fold increases compared to when the cells were cultured with glucose (Fig. 4).

### Conservation of the putative PAH-degrading gene cluster of strain MA-2 in other *Roseobacteraceae* strains

Comprehensive analyses of bacterial genomes in databases revealed that this putative PAH-degrading gene cluster was highly conserved in genomes of five other *Roseobacteraceae* strains that do not belong to *Sagittula* (*Salipiger aestuarii* DSM 22011^T^, *S. aestuarii* 357, *S. pentaromativorans* P9^T^, *Puniceibacterium* sp. IMCC21224, and *Oceanicola* sp. MCTG156(1a)), exhibiting 77%–100% amino acid identities and the same order and orientations of the genes (Fig. 3; Table S2), while this gene cluster was not found in all other four *Sagittula* strains in public databases. Furthermore, potential homologs (with 35%–78% amino acid identities) of the genes contained in this gene cluster were also identified in genomes across different *Roseobacteraceae* genera (*Salipiger*, *Confluentimicrobium*, *Celeribacter*, and *Rhodovulum*; Fig. 2), forming gene clusters with different gene orders and orientations compared to the gene cluster in strain MA-2 (Fig. 3; Table S2). These bacterial strains include *C. indicus* P73^T^, a known PAH-degrading *Roseobacteraceae* strain, and the gene cluster found in this strain was previously characterized to be responsible for the biodegradation of PAHs including phenanthrene (23). Among the *Roseobacteraceae* strains identified here, *Salipiger aestuarii* 357, *Celeribacter persicus* DSM 100434^T^, and *Rhodovulum* sp. NI22 were previously reported to have the ability to grow on PAHs (naphthalene, phenanthrene, and fluorene) as sole carbon sources, and some other strains were also reported for their potential for PAH biodegradation (Table 2 and references herein; (22, 26–32)). Based on these findings, the putative PAH-degrading gene cluster identified in the strain MA-2 genome was considered to be shared within different *Roseobacteraceae* genera, exhibiting ANI values ranging from 78.1% to 80.7% with strain MA-2 (Table S3).

**TABLE 2:** Summary of the bacterial strains found to possess the putative PAH-degrading gene cluster

Bacteria	Isolation source	PAH biodegradation capability confirmed through bacterial cultivation tests	IMG genome ID (complete/draft)	Reference
Type A				
*Sagittula* sp. MA-2	Coastal seawater, Japan	Growth on phenanthrene as the sole carbon source	8019782355 (complete)	(24), this study
*Salipiger aestuarii* DSM 22011^T^	Tidal flat, South Korea	–^*a*^	2615840712 (draft)	(26)
*Salipiger aestuarii* 357	Oil-polluted beach sand, Spain	Growth on naphthalene as sole carbon source	2513237353 (draft)	(27) (28)
*Salipiger pentaromativorans* P9^T^	Mangrove sediment, China	Biodegradation of benzo[*a*]pyrene on agar plates	8047621563 (draft)	(29)
*Puniceibacterium* sp. IMCC21224	Coastal seawater, Antarctica	–	2648501741 (draft)	–
*Oceanicola* sp. MCTG156(1a)	Coastal seawater, Scotland	Biodegradation of phenanthrene on agar plates	2579779169 (draft)	(30)
Type B				
*Confluentimicrobium naphthalenivorans* DSM 105040^T^	Tidal-flat, South Korea	Biodegradation of naphthalene in seawater	2830026743 (draft)	(31)
*Celeribacter persicus* DSM 100434^T^	Mangrove forests, Iranian Persian Gulf	Growth on fluorene and phenanthrene as the sole carbon source	2734482187 (draft)	(22)
*Salipiger abyssi* SY10-16	Shallow sea sediment, China	–	3001913768 (draft)	–
*Rhodovulum* sp. NI22	Coastal seawater, Florida, USA	Growth on naphthalene as the sole carbon source	2645727736 (draft)	(32)
*Thioclava dalianensis* DLFJ1-1^T^	Surface seawater, China	–	2585427678 (draft)	(33)
*Neorhizobium petrolearium* DSM 26482^T^	Oil-contaminated soil, China	Growth on phenanthrene as the sole carbon source	2738541324 (draft)	(34)
*Martelella* sp. AD-3	Highly saline petroleum-contaminated soil, China	Growth on phenanthrene as the sole carbon source	2671180917 (complete)	(35)
Type C				
*Celeribacter indicus* P73^T^	Deep-sea sediment from the Indian Ocean	Growth on fluoranthene as the sole carbon source	2693429899 (draft)	(23)

^*a*^ –, unconfirmed.

This gene cluster was further categorized into three sub-classes: types A, B, and C, based on the order and orientations of the genes (Fig. 3). Among these three sub-classes, the type B cluster was also found in genomes from at least three strains from different families from *Roseobacteraceae*, i.e., *Thioclava dalianensis* DLFJ1-1^T^ from *Paracoccaceae* (33) and *Neorhizobium petrolearium* DSM 26482^T^ and *Martelella* sp. AD-3 from *Rhizobiaceae* (Fig. 3; Table 2). These two *Rhizobiaceae* strains were both isolated from petroleum-contaminated soil environments and reported to be capable of growing on phenanthrene as the sole carbon source (34, 35). The type C cluster was only identified in the genome of *C. indicus* P73^T^, of which genes encoding GDO, Nag enzymes, and TRAP transporters were separately located from other genes, thus forming two clusters (Fig. 3).

### Phylogenetic characterization of ARHDs conserved in *Roseobacteraceae*

**Fig 5:**
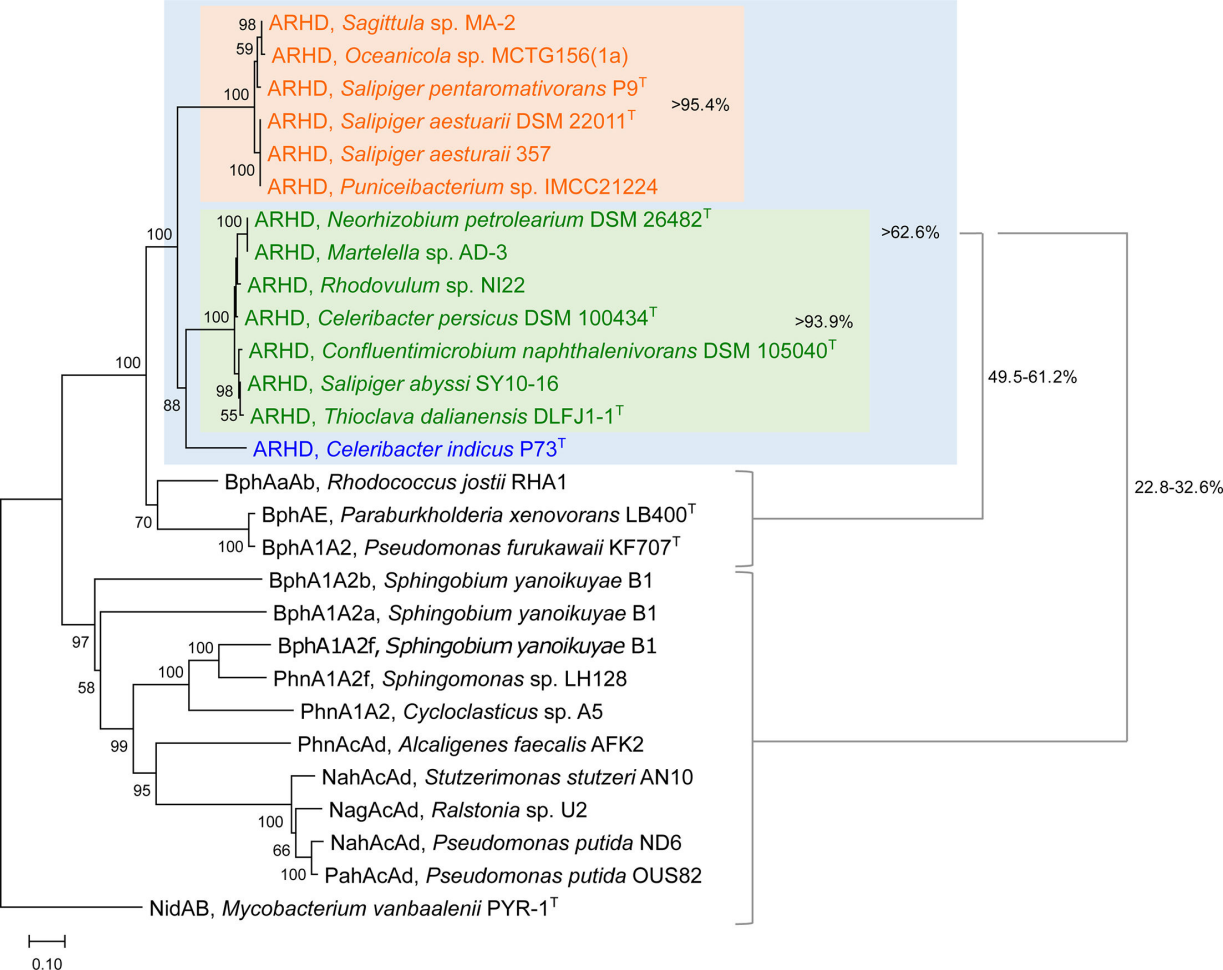
Neighbor-joining phylogenetic tree of concatenated sequences of the large and small subunits of ARHDs from *Roseobacteraceae* (orange, type A; green, type B; and blue, type C as shown in Fig. 3) and other representative PAH-degrading bacterial strains. NidAB (PAH dioxygenase) from *Mycobacterium vanbaalenii* PYR-1^T^ is included as an outgroup. The tree was created with 1,000 bootstrap iterations, and values below 50% are not shown.

Phylogenetic characterization of the putative ARHD enzymes found in *Roseobacteraceae* genomes, based on the concatenated amino acid sequences of the large and small subunits, indicated that the ARHDs from all the types A, B, and C gene clusters shared >93% identities within the same type and >62% identities between different types (Fig. 5). Further comparisons with experimentally verified PAH-degrading ARHD enzymes from selected representative bacteria revealed that the ARHDs from *Roseobacteraceae* exhibited 49%–61% identities to the biphenyl dioxygenases (BphAaAb/BphAE/BphA1A2) from polychlorinated biphenyl-degrading bacteria (*R. jostii* RHA1, *Paraburkholderia xenovorans* LB400^T^, and *Pseudomonas furukawaii* KF707^T^) (25, 36, 37) and formed a phylogenetically distant group from the other ARHD enzymes (up to 33% identities; Fig. 5).

### Hypothetical phenanthrene biodegradation pathway in strain MA-2

In the strain MA-2 culture grown on 50 mg L^−1^ phenanthrene, >99% of the phenanthrene had been consumed by day 7 of incubation, as confirmed by HPLC analysis. Subsequent high-resolution mass-spectrometry (HRMS) profiling of phenanthrene biotransformation products detected and identified eight products (I–VIII), and as shown in Table 3, the theoretical masses and observed masses were in high agreement (mass accuracy < 3.0 ppm in all cases; Table 3). Among these products, the identities of product II (1-hydroxy-2-naphthoic acid, 1H2N), product III (1,2-dihydroxynaphthalene), product VII (2-carboxybenzaldehyde), and product VIII (phthalic acid) were further confirmed by comparing their retention times with authentic standards (Table 3; Fig. S2). In addition, the structures of product II (1H2N) and product VI (2-carboxybenzalpyruvic acid) were further examined and confirmed by HRMS high-energy collisional dissociation (HCD) (Fig. S3).

**TABLE 3:** Phenanthrene biotransformation products in the strain MA-2 cultures identified by liquid chromatography-high-resolution mass spectrometry analyses

Product	Proposed identity	Chemical formula	[M – H]^−^	Retention time (min)	Retention time of standards (min)	Observed *m*/*z*	Theoretical *m*/*z*	Mass error (ppm)
I	*o*-Hydroxynaphthyl-α-oxobutenoates	C_14_H_9_O_4_^−^	241	6.0	–^*a*^	241.0506	241.0506	−0.1531
II	1-Hydroxy-2-naphthoic acid	C_11_H_7_O_3_^−^	187	5.9	5.8	187.0396	187.0401	−2.4626
III	1,2-Dihydroxynaphthalene	C_10_H_7_O_2_^−^	159	6.4	6.5	159.0451	159.0452	−0.4811
IV	2-Hydroxybenzalpyruvic acid	C_10_H_7_O_4_^−^	191	3.7	–	191.0349	191.0350	−0.6445
V	Salicylaldehyde	C_7_H_5_O_2_^−^	121	4.5	–	121.0293	121.0295	−1.5594
VI	2-Carboxybenzalpyruvic acid	C_11_H_7_O_5_^-^	219	5.9	–	219.0303	219.0299	1.7901
VII	2-Carboxybenzaldehyde	C_8_H_5_O_3_^-^	149	5.8	5.7	149.0242	149.0244	−1.3027
VIII	Phthalic acid	C_8_H_5_O_4_^-^	165	4.4	4.1	165.0191	165.0193	−1.1700

^*a*^ –, not analyzed.

**Fig 6:**
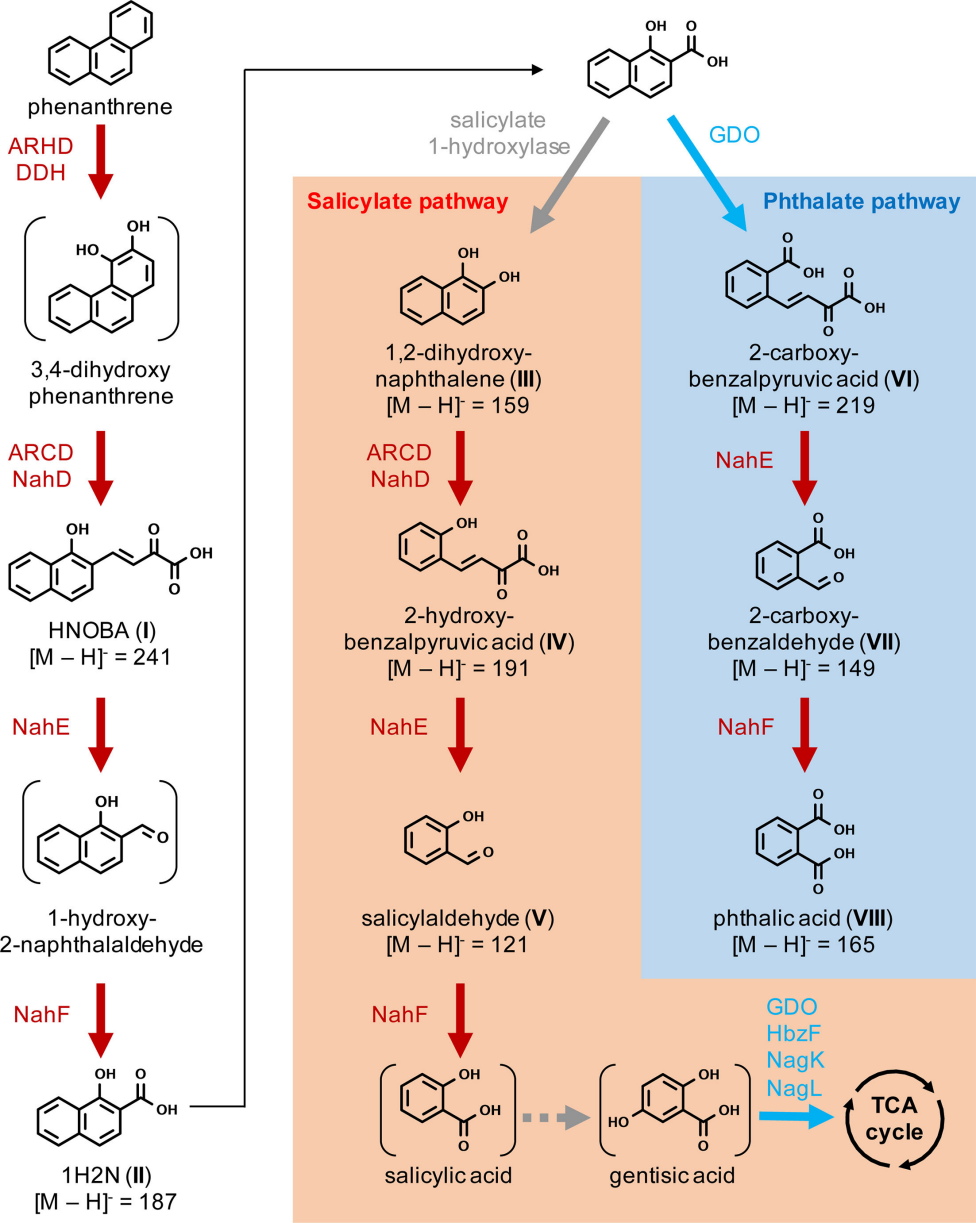
Proposed phenanthrene biodegradation pathways in strain MA-2 based on genomic information and metabolites I–VIII that were identified through liquid chromatography high-resolution mass spectrometry analyses. Enzymes proposed to catalyze each reaction are indicated with colors corresponding to the gene map shown in Fig. 3.

Based on genomic information and the identification of biotransformation products, the phenanthrene biodegradation pathway in strain MA-2 was proposed (Fig. 6). ARHD and DDH are predicted to be responsible for the initial oxidation of phenanthrene, followed by the ring cleavage of 3,4-dihydroxyphenanthrene by ARCD and transformation into *o*-hydroxynaphthyl-α-oxobutenoates (HNOBA, product I) by NahD. HNOBA is proposed to be further transformed by NahE and NahF into 1-hydroxy-2-naphthoic acid (product II). After 1H2N, two separate pathways appeared to occur, known as the salicylate pathway and the phthalate pathway (Fig. 6). The salicylate pathway is initiated by the transformation of 1H2N into 1,2-dihydroxynaphthalene (product III). The enzyme catalyzing this reaction was not determined from the predicted proteins of the gene cluster. Instead, a gene found in the main chromosome (IMG gene ID 8019784713) was annotated as encoding salicylate 1-hydroxylase (EC 1.14.13.1), which was previously characterized as converting not only salicylic acid into catechol but also the structurally analogous 1H2N into 1,2-dihydroxynaphthalene (38, 39), and it is proposed to be responsible for the transformation of 1H2N into 1,2-dihydroxynaphthalene in strain MA-2. The enzymes responsible for the transformation of 3,4-dihydroxyphenanthrene into 1H2N putatively also transform 1,2-dihyroxynaphthalene into salicylic acid via 2-hydroxybenzalpyruvic acid (product IV) and salicylaldehyde (product V). The downstream degradation pathway after salicylic acid is unknown; enzymes for gentisic acid degradation may be involved in the downstream process into the TCA cycle. On the other hand, the presence of 2-carboxybenzalpyruvic acid (product VI), 2-carboxybenzaldehyde (product VII), and phthalic acid (product VIII) suggested the occurrence of ring cleavage of 1H2N and transformation through the phthalic acid pathway. Ring cleavage of 1H2N is hypothetically catalyzed by gentisate 1,2-dioxygenase, which catalyzes the ring cleave of gentisic acid that has an analogous structure to 1H2N. The known genes for phthalic acid degradation, such as phthalate 4,5-dioxygenase (EC 1.14.12.7), were not found in the strain MA-2 genome. Consistently, additional growth tests indicated that strain MA-2 was incapable of growing on phthalic acid as the sole carbon source; however, it showed clear growth on salicylic acid and gentisic acid (data not shown).

### Predictions of protein structures and substrate binding sites of ARHD, ARCD, and GDO encoded by strain MA-2

To estimate the functions of the enzymes encoded in the putative PAH-degrading gene cluster of strain MA-2, protein structural predictions and substrate docking simulations were further carried out (Fig. S4). According to the simulation results, the theoretically most favorable binding site for phenanthrene to the ARHD-LSU is located at the putative active site close to the catalytic mononuclear iron, which is predicted to be coordinated by residues of two histidines and a single aspartic acid. A similar model was previously demonstrated in model ARHD (naphthalene 1,2-dioxygenase) from a *Pseudomonas* sp. that biotransformed phenanthrene ([40]; deposited in Protein Data Bank, PDB, under ID 2HMK). Structural prediction of the ARCD and docking simulation with 3,4-dihydroxyphenanthrene revealed that the substrate favorably binds into a pocket located close to the catalytic mononuclear iron, which is coordinated by residues of two histidines and a single glutamic acid, of which a similar model was previously reported in the model ARCD (catechol 2,3-dioxygenase) of *Pseudomonas* spp. ([41, 42]; PDB ID 1MPY). The GDO of strain MA-2, which is hypothesized to catalyze the ring cleavage of 1-hydroxy-2-naphthoic acid in addition to gentisic acid (Fig. 6), was subjected to substrate docking simulations with gentisic acid and 1H2N. The results indicated that both substrates preferably bind (simulated binding energies: −4.711 kcal/mol for gentisic acid and −5.020 kcal/mol for 1H2N) into the binding pocket closely located at the catalytic mononuclear iron, coordinated by three histidines, of which a similar model was previously reported in GDO (NagI) from *Escherichia coli* ([43]; PDB ID 2D40).

## DISCUSSION

The enormous physiological diversity of *Roseobacteraceae* and their ability to adapt to diverse environmental conditions are considered to be attributed to their genomic plasticity, in other words, due to the frequent acquisition of ecologically relevant genes via horizontal gene transfers (18, 44, 45). A marine bacterial isolate belonging to *Roseobacteraceae*, *Sagittula* sp. strain MA-2, with eight circular plasmids in its genome fully sequenced, represents an adequate model to study the functional genes potentially exchanged among *Roseobacteraceae*. In the current study, strain MA-2 was proven to be capable of biodegrading phenanthrene and growing on it as the sole carbon source, which was not reported in other *Sagittula* strains in the past. Consistent with this, a gene cluster putatively responsible for phenanthrene biotransformation was identified on one of the eight plasmids of strain MA-2, and the transcriptional levels of the functional genes in this cluster were observed to be significantly increased during phenanthrene biodegradation (Fig. 4). This gene cluster was not found in the genomes of other *Sagittula* strains in databases, including a complete genome from *Sagittula* sp. strain P11 (46). Among these *Sagittula* strains, *S. stellata* strain E-37 was claimed to be capable of co-metabolically biodegrading pyrene and phenanthrene, although this strain was also confirmed to be incapable of growing on these PAHs as sole carbon sources (21). These facts further suggested that the putative PAH-degrading gene cluster uniquely possessed by strain MA-2 enabled this strain to biodegrade phenanthrene and utilize it as the growth substrate.

Comprehensive analyses of bacterial genomes in the databases further revealed that gene clusters potentially homologous to this newly found putative PAH-degrading gene cluster are found broadly across *Roseobacteraceae* genera. Similar to the *Sagittula* strains, this gene cluster appeared to be heterogeneously distributed within each different genus (Fig. 2), suggesting that the gene cluster was introduced into these different *Roseobacteraceae* genera via horizontal gene transfers. Furthermore, one type (type B) of the three sub-classes identified within this gene cluster was found in at least three non-*Roseobacteraceae* strains, i.e., strains belonging to *Paracoccaceae* and *Rhizobiaceae*. Among these three strains, *Thioclava dalianensis* DLFJ1-1^T^ (*Paracoccaceae*) is a marine bacterium isolated from surface seawater (33), and its capability of PAH degradation has not been investigated. Although the other two strains belong to *Rhizobiaceae* and both originated from soil environments, *Martelella* sp. AD-3 was characterized as a halophilic bacterium. It was isolated from petroleum-contaminated soil with high salinity and was capable of biodegrading phenanthrene under 15% salinity (35). Another strain, *N. petrolearium* DSM 26482^T^, was also previously reported to be capable of growing on phenanthrene under 8% salinity (34). These observations suggest that enzymes encoded in this gene cluster have adapted to function under saline circumstances. The type C gene cluster was identified only in the genome of a PAH-degrading bacterium *C. indicus* P73^T^, and the functions of the enzymes encoded in this gene cluster in fluoranthene biodegradation were previously proposed (23). Intriguingly, this strain was isolated from deep-sea sediment, while none of the other *Roseobacteraceae* strains studied here originated from deep-sea environments (Table 2). This fact further raises the hypothesis that the type C gene cluster has been conserved and evolved among bacteria in deep-sea environments, while the other two types have been exchanged among bacteria in coastal marine environments and even certain terrestrial environments with high salinity, which requires larger comprehensive bacterial (meta)genomic investigations, especially from deep-sea environments, to prove this hypothesis.

1-Hydroxy-2-naphthoic acid is known as a common intermediate in bacterial phenanthrene biodegradation, and the biotransformation of 1H2N has been repeatedly characterized as the limiting step preventing the growth of naphthalene-degrading bacteria on phenanthrene (38). The cell yield of strain MA-2 grown on phenanthrene appeared to be limited compared to other phenanthrene-degrading bacterial strains in the literature, such as *Martelella* sp. AD-3, which was reported to exceed 1.0 × 10^9^ cells mL^−1^ at the stationary phase when grown on phenanthrene (35). This growth limitation may have been caused by transient accumulation of downstream compounds such as 1H2N. Based on the evidence obtained through HRMS profiling of phenanthrene biotransformation products, strain MA-2 appeared to biodegrade phenanthrene via two separated, concurrent pathways through 1H2N, namely the salicylate pathway and the phthalate pathway (47, 48) (Fig. 6). A similar phenomenon has been observed in other bacterial genera, including *Sphingobium*, *Pseudomonas*, and *Mycobacterium* (49–51). In Gram-negative bacteria, while enzymes responsible for the salicylate pathway biodegradation of phenanthrene have been extensively investigated, such as Nah enzymes as representatives, enzymes involved in the phthalate pathway have not been sufficiently characterized in the past. A known enzyme responsible for the key reaction of the phthalate pathway, i.e., the ring cleavage of 1H2N, is 1-hydroxy-2-naphthoate 1,2-dioxygenase PhdI (EC 1.13.11.38). It was first identified in a Gram-positive marine bacterium *Nocardioides* sp. strain KP7 (52) and is widely conserved in PAH-degrading *Mycobacterium* strains (10, 53). In strain MA-2, the ring cleavage of 1H2N is hypothetically catalyzed by the enzyme annotated as gentisate 1,2-dioxygenase (EC 1.13.11.4; Fig. 6; Fig. S4), which exhibits 26.3% amino acid identity to PhdI from *Nocardioides* sp. strain KP7. In fact, PhdI and GDO were previously characterized as sharing similar structures that conserve a two-domain cupin composition (54, 55), thus further supporting our hypothesis that GDO in strain MA-2 is responsible for the ring cleavage of 1H2N. The *in silico* protein structural predictions and substrate docking simulations conducted in this study provided evidence supporting the hypothetical functions of targeted proteins without the need for protein purification or establishment of bacterial mutants. However, researchers previously have pointed to the inaccuracies and imperfections of deep learning-based structural predictions in protein folding (56, 57). Therefore, further experiments using purified proteins are required to fully prove their functions and elucidate the detailed phenanthrene biodegradation pathway in strain MA-2.

Although strain MA-2 appeared to biodegrade phenanthrene down to phthalic acid, genes encoding proteins related to the known enzymes to biotransform phthalic acid were not determined in its genome, and it appeared incapable of utilizing phthalic acid as a growth substrate. Interestingly, in the phenanthrene-enriched bacterial consortium where strain MA-2 was isolated, the genus *Thalassospira* was identified as the dominant consortium member (Mori et al., unpublished data), and an isolate of *Thalassospira* obtained from this consortium, strain GO-4, was previously characterized to grow on phthalic acid and its precursor 2-carboxybenzaldehyde in the consortium, while incapable of degrading phenanthrene (58). Thus, *Thalassospira* likely grew in the consortium under metabolic dependency on the phenanthrene biotransformation products released from co-existing *Sagittula* and contributed to the synergistic biodegradation of phenanthrene. These observations enhance our understanding of multi-species microbial PAH biodegradation in marine ecosystems, where the members of *Roseobacteraceae* occasionally acquire PAH-degrading ability and potentially play pioneering roles in PAH biodegradation. This enables other microorganisms to grow by providing more bioavailable hydrocarbon substrates and may support the ecosystem.

In conclusion, this study provides genomic insights into a phenanthrene-degrading *Roseobacteraceae* isolate, *Sagittula* sp. strain MA-2, identifying a putative PAH-degrading gene cluster on one of its eight plasmids. Gene clusters potentially homologous to this newly identified cluster are found widely but heterogeneously distributed among *Roseobacteraceae* strains originating from coastal and deep-sea environments. Although the current investigation was limited to a single bacterial strain and remains inferential based on genomic evidence, the proposed phenanthrene biodegradation ability in strain MA-2 may be potentially conserved in other *Roseobacteraceae* strains that possess the gene cluster and shall require future investigation. Knowledge obtained in this study may be applied to improve our understanding of biotechnologically useful bacteria and their enzymes that contribute to the remediation of polluted marine environments or high-salinity wastewater. Additionally, further investigations into the functional genes present on the plasmids of strain MA-2 may allow us to discover yet-to-be-known physiological abilities exchanged and conserved among *Roseobacteraceae*.

## MATERIALS AND METHODS

### Chemicals

Phenanthrene (98% purity) was purchased from Sigma-Aldrich (St. Louis, MO, USA). Gentisic acid (98% purity), phthalic acid (98% purity), salicylic acid (>99.5% purity), *N,N*-dimethylformamide (DMF; >99% purity), and methanol (LC-MS grade) were purchased from Wako Chemical (Osaka, Japan). 1-Hydroxy-2-naphthoic acid (>97% purity) was purchased from Kanto Chemical Co. (Tokyo, Japan). 1,2-Dihydroxynaphthalene (>95% purity) and 2-carboxybenzaldehyde (>98% purity) were purchased from Tokyo Chemical Industries (Tokyo, Japan).

### Bacterial isolation and growth condition

*Sagittula* sp. strain MA-2 was isolated from a phenanthrene-degrading marine bacterial consortium that originated from the coast of Nojima, Yokohama, Japan (24), as part of an ongoing study investigating aromatic hydrocarbon-degrading marine microorganisms in the urban coastal area (58, 59). The strain MA-2 cells were cultured with 50 mg L^−1^ gentisic acid as the sole carbon source in artificial sea water medium (60), with rotary shaking at 150 rpm in the dark at 30°C, which was repeatedly sub-cultured once per month. The growth capability of strain MA-2 on different carbon sources (phenanthrene, salicylic acid, or phthalic acid; in DMF prior to addition) was tested by culturing with 50 mg L^−1^ of the selected substrates for 7 days and subsequently transferring bacterial cells into a new medium containing the same carbon sources. Bacterial growth was evaluated through visual inspection of turbidity and microscopic cell counting using SYTO 13 nucleic acid staining (Thermo Fisher Scientific, Waltham, MA, USA) as described previously (61).

### Genomic investigations of strain MA-2 and comparative genomic analyses

The complete genome sequence of strain MA-2 was announced in a previous report, which included detailed methodological information on sequencing analyses and data processing (24). The genomic DNA extraction for sequencing analyses was conducted concurrently with the bacterial growth tests described above to maintain consistency between their genomic features and growth capabilities. The ANI between the strain MA-2 genome and reference bacterial genomes was determined using fastANI version 1.33 (62). Aromatic hydrocarbon biodegradation functional gene candidates in the strain MA-2 genome were identified through orthology inference with experimentally verified enzymes included in the Hydrocarbon Aerobic Degradation Enzymes and Genes database (63) using SonicParanoid version 1.3.8 (64).

### Transcriptional analysis of putative PAH-degrading genes by RT-qPCR

Reverse transcription-quantitative PCR was conducted to investigate the transcriptional levels of genes encoding putative PAH-degrading enzymes of strain MA-2 under exposure to the PAH, phenanthrene. Strain MA-2 cells pre-grown on 10 mM glucose were collected during the exponential growth phase (at an OD_600_ of 0.10) by centrifugation, washed with fresh ASW medium, and resuspended in the same volume of fresh ASW medium. The resuspended cells were divided into multiple flasks and further incubated with either 50 mg L^−1^ phenanthrene or 10 mM glucose (as the control) as the sole carbon source. After 24 hours, bacterial RNA was extracted using the NucleoSpin RNA kit (Macherey-Nagel, Düren, Germany), and cDNA was synthesized through reverse transcription using PrimeScript reverse transcriptase (Takara Bio, Otsu, Japan). Quantitative PCR was performed on a qTOWER^3^ G thermal cycler (Analytik Jena, Jena, Germany) using innuMIX qPCR SyGreen Standard (Analytik Jena) and primer pairs for the amplification of genes encoding the large subunit of ARHD (ARHD-LSU), ARCD, GDO, or a replication initiation protein RepC—the *repC* gene, located on the same plasmid as the putative PAH-degrading gene cluster, was selected as the internal reference. The newly designed primer pairs used for RT-qPCR are listed in Table S4. Transcriptional levels of the targeted genes were determined using the 2^-ΔΔCt^ method.

### Identification of phenanthrene biotransformation products of strain MA-2 by LC-HRMS

Phenanthrene biotransformation products produced by strain MA-2 were extracted from the bacterial cultures that grew on 50 mg L^−1^ phenanthrene for 4 and 7 days, with equal volumes of ethyl acetate at pH 7 and pH 2 by liquid-liquid extraction based upon previous PAH extraction methods (65). The extract residues were resuspended in 1 mL of methanol and chromatographically separated by using a Thermo Scientific Ultimate 3000 LC coupled to a Thermo Scientific model Q Exactive Focus hybrid quadrupole-Orbitrap mass spectrometer (liquid chromatography electrospray ionization high-resolution mass-spectrometry, LC/ESI-HRMS) under conditions according to Sakai et al. (66) by using an XSelect CSH C18 column (4.6 mm i.d. × 150 mm, 3.5 µm particle size; Waters, Milford, MA, USA) that was in-line with a Security Guard Cartridge System pre-column fitted with a widepore C18 cartridge (Phenomenex, Torrance, CA, USA). The HRMS was equipped with a heated electrospray ionization source and operated in negative electrospray ionization mode, and the probe parameters were set for the sheath gas, auxiliary gas, and sweep gas (all nitrogen) as follows: 45 arbitrary units, 10 arbitrary units, and 0 arbitrary units, respectively. The temperature of the probe heater was 300°C, the capillary temperature was 350°C, the ion spray voltage was 2.0 kV, and the S-lens RF level was 50. In full scan MS mode, ions were scanned over the range from *m/z* 115 to *m/z* 500, data were acquired at a resolution of 35,000 FWHM, and the AGC was set to 1 × 10^6^ ions for a maximum injection time of 120 ms. Precursor ions were filtered by the quadrupole in a 0.4 *m/z* isolation window and fragmented using HCD with stepped collision energies of 10 and 20 eV (67). Thermo Scientific Xcalibur software version 4.1 was used for data acquisition and processing.

### Protein structure prediction and substrate docking simulation

Three-dimensional structures of selected proteins encoded in the strain MA-2 genome were predicted by using AlphaFold2 (68). The positions of nuclear metal ions within these proteins were further predicted using Metal3D (69). Molecular docking simulations of selected substrates to the predicted protein models were conducted using the AutoDock Vina program (70, 71) on Chimera after structural optimizations of the target proteins and ligands with the Dock Prep module. The best docking models, determined by the lowest binding energy, were simulated and visualized using the PyMOL Molecular Graphics System (version 2.0; Schrödinger, LLC).

## Data Availability

The complete genome sequence of strain MA-2 has been deposited in NCBI GenBank under accession numbers CP126145-CP126153 and the IMG database under Genome ID 8019782355.

## SUPPLEMENTAL MATERIAL

Figures S1 to S4 spectrum.01074-24-s0001.docx Fig. S1: Gene map of the strain MA-2 plasmid p6. Fig. S2: Results of LC-HRMS of strain MA-2 extract. Fig. S3: Results of HCD of phenanthrene biotransformation products in strain MA-2. Fig. S4: Predicted 3D structure of the selected enzymes in strain MA-2.

Table S1 spectrum.01074-24-s0002.xlsx List of predicted aromatic hydrocarbon degradation genes.

Table S2 spectrum.01074-24-s0003.xlsx List of functional genes in the PAH-degrading gene cluster.

Table S3 spectrum.01074-24-s0004.xlsx Average nucleic acid identity among *Sagittula* sp. MA-2 and other bacteria.

Table S4 spectrum.01074-24-s0005.xlsx Primers used for the RT-qPCR analysis.

ASM does not own the copyrights to Supplemental Material that may be linked to, or accessed through, an article. The authors have granted ASM a non-exclusive, world-wide license to publish the Supplemental Material files. Please contact the corresponding author directly for reuse.

## References

[1] Khara P, Roy M, Chakraborty J, Ghosal D, Dutta TK. 2014. Functional characterization of diverse ring-hydroxylating oxygenases and induction of complex aromatic catabolic gene clusters in Sphingobium sp. PNB. FEBS Open Bio 4:290–300. doi:10.1016/j.fob.2014.03.001 PMC4048848 24918041

[2] Parales RE, Resnick SM. 2004. Aromatic hydrocarbon dioxygenases BT - biodegradation and bioremediation, p 175–195. In Singh A, Ward OP (ed),. Springer, Berlin Heidelberg, Berlin, Heidelberg.

[3] Harayama S, Rekik M. 1989. Bacterial aromatic ring-cleavage enzymes are classified into two different gene families. J Biol Chem 264:15328–15333. doi:10.1016/S0021-9258(19)84830-5 2670937

[4] Peng R-H, Xiong A-S, Xue Y, Fu X-Y, Gao F, Zhao W, Tian Y-S, Yao Q-H. 2008. Microbial biodegradation of polyaromatic hydrocarbons. FEMS Microbiol Rev 32:927–955. doi:10.1111/j.1574-6976.2008.00127.x 18662317

[5] Li W, Shi J, Wang X, Han Y, Tong W, Ma L, Liu B, Cai B. 2004. Complete nucleotide sequence and organization of the naphthalene catabolic plasmid pND6-1 from Pseudomonas sp. strain ND6. Gene 336:231–240. doi:10.1016/j.gene.2004.03.027 15246534

[6] Kiyohara H, Torigoe S, Kaida N, Asaki T, Iida T, Hayashi H, Takizawa N. 1994. Cloning and characterization of a chromosomal gene cluster, pah, that encodes the upper pathway for phenanthrene and naphthalene utilization by Pseudomonas putida OUS82. J Bacteriol 176:2439–2443. doi:10.1128/jb.176.8.2439-2443.1994 8157614 PMC205370

[7] Pinyakong O, Habe H, Omori T. 2003. The unique aromatic catabolic genes in sphingomonads degrading polycyclic aromatic hydrocarbons (PAHs). J Gen Appl Microbiol 49:1–19. doi:10.2323/jgam.49.1 12682862

[8] Maeda AH, Nishi S, Hatada Y, Ohta Y, Misaka K, Kunihiro M, Mori JF, Kanaly RA. 2020. Chemical and genomic analyses of polycyclic aromatic hydrocarbon biodegradation in Sphingobium barthaii KK22 reveals divergent pathways in soil sphingomonads. International Biodeterioration & Biodegradation 151:104993. doi:10.1016/j.ibiod.2020.104993

[9] Schuler L, Jouanneau Y, Chadhain SMN, Meyer C, Pouli M, Zylstra GJ, Hols P, Agathos SN. 2009. Characterization of a ring-hydroxylating dioxygenase from phenanthrene-degrading Sphingomonas sp. strain LH128 able to oxidize benz[a]anthracene. Appl Microbiol Biotechnol 83:465–475. doi:10.1007/s00253-009-1858-2 19172265

[10] Stingley RL, Khan AA, Cerniglia CE. 2004. Molecular characterization of a phenanthrene degradation pathway in Mycobacterium vanbaalenii PYR-1. Biochem Biophys Res Commun 322:133–146. doi:10.1016/j.bbrc.2004.07.089 15313184

[11] Pagnout C, Frache G, Poupin P, Maunit B, Muller J-F, Férard J-F. 2007. Isolation and characterization of a gene cluster involved in PAH degradation in Mycobacterium sp. strain SNP11: expression in Mycobacterium smegmatis mc(2)155. Res Microbiol 158:175–186. doi:10.1016/j.resmic.2006.11.002 17258432

[12] Liang C, Huang Y, Wang H. 2022. ltigtpahE/i, a functional marker gene for polycyclic aromatic hydrocarbon-degrading bacteria. Appl Environ Microbiol:e02399-18. doi:10.1128/AEM.02399-18 30478232 PMC6344622

[13] Mori JF, Kanaly RA. 2020. Multispecies diesel fuel biodegradation and niche formation are ignited by pioneer hydrocarbon-utilizing proteobacteria in a soil bacterial consortium. Appl Environ Microbiol 87:e02268-20. doi:10.1128/AEM.02268-20 33067200 PMC7755252

[14] Duran R, Cravo-Laureau C. 2016. Role of environmental factors and microorganisms in determining the fate of polycyclic aromatic hydrocarbons in the marine environment. FEMS Microbiol Rev 40:814–830. doi:10.1093/femsre/fuw031 28201512 PMC5091036

[15] Haritash AK. 2020. A comprehensive review of metabolic and genomic aspects of PAH-degradation. Arch Microbiol 202:2033–2058. doi:10.1007/s00203-020-01929-5 32506150

[16] Kasai Y, Shindo K, Harayama S, Misawa N. 2003. Molecular characterization and substrate preference of a polycyclic aromatic hydrocarbon dioxygenase from Cycloclasticus sp. strain A5. Appl Environ Microbiol 69:6688–6697. doi:10.1128/AEM.69.11.6688-6697.2003 14602629 PMC262276

[17] Bosch R, García-Valdés E, Moore ER. 1999. Genetic characterization and evolutionary implications of a chromosomally encoded naphthalene-degradation upper pathway from Pseudomonas stutzeri AN10. Gene 236:149–157. doi:10.1016/s0378-1119(99)00241-3 10433976

[18] Simon M, Scheuner C, Meier-Kolthoff JP, Brinkhoff T, Wagner-Döbler I, Ulbrich M, Klenk H-P, Schomburg D, Petersen J, Göker M. 2017. Phylogenomics of Rhodobacteraceae reveals evolutionary adaptation to marine and non-marine habitats. ISME J 11:1483–1499. doi:10.1038/ismej.2016.198 28106881 PMC5437341

[19] Liang KYH, Orata FD, Boucher YF, Case RJ. 2021. Roseobacters in a sea of poly- and paraphyly: whole genome-based taxonomy of the family Rhodobacteraceae and the proposal for the split of the “roseobacter clade” Into a novel family, Roseobacteraceae fam. nov. Front Microbiol 12:683109. doi:10.3389/fmicb.2021.683109 34248901 PMC8267831

[20] Zhou H, Zhang S, Xie J, Wei H, Hu Z, Wang H. 2020. Pyrene biodegradation and its potential pathway involving roseobacter clade bacteria. Int Biodeterior Biodegradation 150:104961. doi:10.1016/j.ibiod.2020.104961

[21] Walton JL, Buchan A. 2024. Evidence for novel polycyclic aromatic hydrocarbon degradation pathways in culturable marine isolates. Microbiol Spectr 12:e0340923. doi:10.1128/spectrum.03409-23 38084970 PMC10783047

[22] Shahriari Moghadam M, Ebrahimipour G, Abtahi B, Ghassempour A, Hashtroudi MS. 2014. Biodegradation of polycyclic aromatic hydrocarbons by a bacterial consortium enriched from mangrove sediments. J Environ Health Sci Engineer 12:114. doi:10.1186/s40201-014-0114-6 PMC4243957 25436114

[23] Cao J, Lai Q, Yuan J, Shao Z. 2015. Genomic and metabolic analysis of fluoranthene degradation pathway in Celeribacter indicus P73^T^. Sci Rep 5:7741. doi:10.1038/srep07741 25582347 PMC4291564

[24] Abe M, Kanaly RA, Mori JF. 2023. Genomic analysis of a marine alphaproteobacterium Sagittula sp. strain MA-2 that carried eight plasmids. Mar Genomics 72:101070. doi:10.1016/j.margen.2023.101070 38008530

[25] Iwasaki T, Takeda H, Miyauchi K, Yamada T, Masai E, Fukuda M. 2007. Characterization of two biphenyl dioxygenases for biphenyl/PCB degradation in A PCB degrader, Rhodococcus sp. strain RHA1. Biosci Biotechnol Biochem 71:993–1002. doi:10.1271/bbb.60663 17420585

[26] Park MS, Chung B-S, Lee HJ, Jin HM, Lee S-S, Oh YK, Jeon CO. 2011. Citreicella aestuarii sp. nov., isolated from a tidal flat. Int J Syst Evol Microbiol 61:2595–2599. doi:10.1099/ijs.0.028332-0 21131501

[27] Suarez-Suarez LY, Brunet-Galmes I, Piña-Villalonga JM, Christie-Oleza JA, Peña A, Bennasar A, Armengaud J, Nogales B, Bosch R. 2012. Draft genome sequence of Citreicella aestuarii strain 357, a member of the Roseobacter clade isolated without xenobiotic pressure from a petroleum-polluted beach. J Bacteriol 194:5464–5465. doi:10.1128/JB.01261-12 22965089 PMC3457245

[28] Gutiérrez MS, León AJ, Duel P, Bosch R, Piña MN, Morey J. 2021. Effective elimination and biodegradation of polycyclic aromatic hydrocarbons from seawater through the formation of magnetic microfibres. IJMS 22:17. doi:10.3390/ijms22010017 PMC7792786 33375008

[29] Li J, Lai Q, Hu A, Liu H, Qin D, Yang X, Tian Y, Yu C-P. 2023. Salipiger pentaromativorans sp. nov., a polycyclic aromatic hydrocarbon-degrading bacterium isolated from mangrove sediment. Int J Syst Evol Microbiol 73:005716. doi:10.1099/ijsem.0.005716 36790415

[30] Gutierrez T, Whitman WB, Huntemann M, Copeland A, Chen A, Vargese N, Kyrpides NC, Pillay M, Ivanova N, Mikhailova N, Mukherjee S, Stamatis D, Reddy TBK, Ngan CY, Chovatia M, Daum C, Shapiro N, Woyke T. 2017. Genome sequence of Oceanicola sp. strain MCTG156(1a), isolated from a scottish coastal phytoplankton net sample. Genome Announc 5:e00796-17. doi:10.1128/genomeA.00796-17 28798189 PMC5552998

[31] Jeong HI, Jin HM, Jeon CO. 2015. Confluentimicrobium naphthalenivorans sp. nov., a naphthalene-degrading bacterium isolated from sea-tidal-flat sediment, and emended description of the genus Confluentimicrobium Park et al. 2015. Int J Syst Evol Microbiol 65:4191–4195. doi:10.1099/ijsem.0.000561 26311461

[32] Brown LM, Gunasekera TS, Bowen LL, Ruiz ON. 2015. Draft genome sequence of Rhodovulum sp. strain NI22, a naphthalene-degrading marine bacterium. Genome Announc 3:e01475-14. doi:10.1128/genomeA.01475-14 PMC4319574 25614575

[33] Zhang R, Lai Q, Wang W, Li S, Shao Z. 2013. Thioclava dalianensis sp. nov., isolated from surface seawater. Int J Syst Evol Microbiol 63:2981–2985. doi:10.1099/ijs.0.046094-0 23378112

[34] Huang X, Shi J, Cui C, Yin H, Zhang R, Ma X, Zhang X. 2016. Biodegradation of phenanthrene by Rhizobium petrolearium SL-1. J Appl Microbiol 121:1616–1626. doi:10.1111/jam.13292 27614183

[35] Feng T -c., Cui C -z., Dong F, Feng Y -y., Liu Y -d., Yang X -m. 2012. Phenanthrene biodegradation by halophilic Martelella sp. AD-3. J Appl Microbiol 113:779–789. doi:10.1111/j.1365-2672.2012.05386.x 22762374

[36] Denef VJ, Park J, Tsoi TV, Rouillard J-M, Zhang H, Wibbenmeyer JA, Verstraete W, Gulari E, Hashsham SA, Tiedje JM. 2004. Biphenyl and benzoate metabolism in a genomic context: outlining genome-wide metabolic networks in Burkholderia xenovorans LB400. Appl Environ Microbiol 70:4961–4970. doi:10.1128/AEM.70.8.4961-4970.2004 15294836 PMC492332

[37] Taira K, Hirose J, Hayashida S, Furukawa K. 1992. Analysis of bph operon from the polychlorinated biphenyl-degrading strain of Pseudomonas pseudoalcaligenes KF707. J Biol Chem 267:4844–4853. doi:10.1016/S0021-9258(18)42908-0 1537863

[38] Balashova NV, Stolz A, Knackmuss HJ, Kosheleva IA, Naumov AV, Boronin AM. 2001. Purification and characterization of a salicylate hydroxylase involved in 1-hydroxy-2-naphthoic acid hydroxylation from the naphthalene and phenanthrene-degrading bacterial strain Pseudomonas putida BS202-P1. Biodegradation 12:179–188. doi:10.1023/a:1013126723719 11826899

[39] Cho O, Choi KY, Zylstra GJ, Kim Y-S, Kim S-K, Lee JH, Sohn H-Y, Kwon G-S, Kim YM, Kim E. 2005. Catabolic role of a three-component salicylate oxygenase from Sphingomonas yanoikuyae B1 in polycyclic aromatic hydrocarbon degradation. Biochem Biophys Res Commun 327:656–662. doi:10.1016/j.bbrc.2004.12.060 15649397

[40] Ferraro DJ, Okerlund AL, Mowers JC, Ramaswamy S. 2006. Structural basis for regioselectivity and stereoselectivity of product formation by naphthalene 1,2-dioxygenase. J Bacteriol 188:6986–6994. doi:10.1128/JB.00707-06 16980501 PMC1595510

[41] Kita A, Kita S, Fujisawa I, Inaka K, Ishida T, Horiike K, Nozaki M, Miki K. 1999. An archetypical extradiol-cleaving catecholic dioxygenase: the crystal structure of catechol 2,3-dioxygenase (metapyrocatechase) from Pseudomonas putida mt-2. Structure 7:25–34. doi:10.1016/s0969-2126(99)80006-9 10368270

[42] Senda T, Sugiyama K, Narita H, Yamamoto T, Kimbara K, Fukuda M, Sato M, Yano K, Mitsui Y. 1996. Three-dimensional structures of free form and two substrate complexes of an extradiol ring-cleavage type dioxygenase, the BphC enzyme from Pseudomonas sp. strain KKS102. J Mol Biol 255:735–752. doi:10.1006/jmbi.1996.0060 8636975

[43] Adams MA, Singh VK, Keller BO, Jia Z. 2006. Structural and biochemical characterization of gentisate 1,2-dioxygenase from Escherichia coli O157:H7. Mol Microbiol 61:1469–1484. doi:10.1111/j.1365-2958.2006.05334.x 16930152

[44] Petersen J, Frank O, Göker M, Pradella S. 2013. Extrachromosomal, extraordinary and essential--the plasmids of the Roseobacter clade. Appl Microbiol Biotechnol 97:2805–2815. doi:10.1007/s00253-013-4746-8 23435940

[45] Brinkmann H, Göker M, Koblížek M, Wagner-Döbler I, Petersen J. 2018. Horizontal operon transfer, plasmids, and the evolution of photosynthesis in Rhodobacteraceae. ISME J 12:1994–2010. doi:10.1038/s41396-018-0150-9 29795276 PMC6052148

[46] Martínez-Pérez C, Mohr W, Schwedt A, Dürschlag J, Callbeck CM, Schunck H, Dekaezemacker J, Buckner CRT, Lavik G, Fuchs BM, Kuypers MMM. 2018. Metabolic versatility of a novel N_2_ -fixing alphaproteobacterium isolated from a marine oxygen minimum zone. Environ Microbiol 20:755–768. doi:10.1111/1462-2920.14008 29194930

[47] Samanta SK, Chakraborti AK, Jain RK. 1999. Degradation of phenanthrene by different bacteria: evidence for novel transformation sequences involving the formation of 1-naphthol. Appl Microbiol Biotechnol 53:98–107. doi:10.1007/s002530051621 10645629

[48] Prabhu Y, Phale PS. 2003. Biodegradation of phenanthrene by Pseudomonas sp. strain PP2: novel metabolic pathway, role of biosurfactant and cell surface hydrophobicity in hydrocarbon assimilation. Appl Microbiol Biotechnol 61:342–351. doi:10.1007/s00253-002-1218-y 12743764

[49] Yin C, Xiong W, Qiu H, Peng W, Deng Z, Lin S, Liang R. 2020. Characterization of the phenanthrene-degrading Sphingobium yanoikuyae SJTF8 in heavy metal co-existing liquid medium and analysis of its metabolic pathway. Microorganisms 8:946. doi:10.3390/microorganisms8060946 32586023 PMC7355620

[50] Sharma M, Salama E-S, Usman M, Khan A, Arif M, Li X. 2023. Evaluation of aerobic biodegradation of phenanthrene using Pseudomonas turukhanskensis: an optimized study. Biodegradation 34:21–41. doi:10.1007/s10532-022-10002-5 36369603

[51] Sun S, Wang H, Chen Y, Lou J, Wu L, Xu J. 2019. Salicylate and phthalate pathways contributed differently on phenanthrene and pyrene degradations in Mycobacterium sp. WY10. J Hazard Mater 364:509–518. doi:10.1016/j.jhazmat.2018.10.064 30388634

[52] Saito A, Iwabuchi T, Harayama S. 1999. Characterization of genes for enzymes involved in the phenanthrene degradation in Nocardioides sp. KP7. Chemosphere 38:1331–1337. doi:10.1016/s0045-6535(98)00534-7 10070721

[53] Krivobok S, Kuony S, Meyer C, Louwagie M, Willison JC, Jouanneau Y. 2003. Identification of pyrene-induced proteins in Mycobacterium sp. strain 6PY1: evidence for two ring-hydroxylating dioxygenases. J Bacteriol 185:3828–3841. doi:10.1128/JB.185.13.3828-3841.2003 12813077 PMC161579

[54] Dunwell JM, Khuri S, Gane PJ. 2000. Microbial relatives of the seed storage proteins of higher plants: conservation of structure and diversification of function during evolution of the cupin superfamily. Microbiol Mol Biol Rev 64:153–179. doi:10.1128/MMBR.64.1.153-179.2000 10704478 PMC98990

[55] Werwath J, Arfmann H-A, Pieper DH, Timmis KN, Wittich R-M. 1998. Biochemical and genetic characterization of a gentisate 1, 2-dioxygenase from Sphingomonas sp. strain RW5. J Bacteriol 180:4171–4176. doi:10.1128/JB.180.16.4171-4176.1998 9696766 PMC107414

[56] Outeiral C, Nissley DA, Deane CM. 2022. Current structure predictors are not learning the physics of protein folding. Bioinformatics 38:1881–1887. doi:10.1093/bioinformatics/btab881 35099504 PMC8963306

[57] Gomes P, Gomes DEB, Bernardi RC. 2022. Protein structure prediction in the era of AI: Challenges and limitations when applying to in silico force spectroscopy. Front Bioinform 2:983306. doi:10.3389/fbinf.2022.983306 36304287 PMC9580946

[58] Kayama G, Kanaly RA, Mori JF. 2022. Comprehensive genomic characterization of marine bacteria Thalassospira spp. provides insights into their ecological roles in aromatic hydrocarbon-exposed environments. Microbiol Spectr 10:e0314922. doi:10.1128/spectrum.03149-22 36190412 PMC9604089

[59] Kayama G, Kanaly RA, Mori JF. 2022. Complete genome sequence of Thalassospira sp. strain GO-4, a marine bacterium isolated from a phenanthrene-enriched bacterial consortium. Microbiol Resour Announc 11:e0053222. doi:10.1128/mra.00532-22 35867521 PMC9387242

[60] Kester DR, Duedall IW, Connors DN, Pytkowicz RM. 1967. Preparation of artificial seawater. Limnol Oceanogr 12:176–179. doi:10.4319/lo.1967.12.1.0176

[61] Mori JF, Scott JJ, Hager KW, Moyer CL, Küsel K, Emerson D. 2017. Physiological and ecological implications of an iron- or hydrogen-oxidizing member of the Zetaproteobacteria, Ghiorsea bivora, gen. nov., sp. nov. ISME J 11:2624–2636. doi:10.1038/ismej.2017.132 28820506 PMC5649172

[62] Jain C, Rodriguez-R LM, Phillippy AM, Konstantinidis KT, Aluru S. 2018. High throughput ANI analysis of 90K prokaryotic genomes reveals clear species boundaries. Nat Commun 9:5114. doi:10.1038/s41467-018-07641-9 30504855 PMC6269478

[63] Rojas-Vargas J, Castelán-Sánchez HG, Pardo-López L. 2023. HADEG: a curated hydrocarbon aerobic degradation enzymes and genes database. Comput Biol Chem 107:107966. doi:10.1016/j.compbiolchem.2023.107966 37778093

[64] Cosentino S, Iwasaki W. 2019. SonicParanoid: fast, accurate and easy orthology inference. Bioinformatics 35:149–151. doi:10.1093/bioinformatics/bty631 30032301 PMC6298048

[65] Izawa M, Sakai M, Mori JF, Kanaly RA. 2021. Cometabolic benzo[a]pyrene biotransformation by Sphingobium barthaii KK22 proceeds through the kata-annelated ring and 1-pyrenecarboxylic acid to downstream products. J Hazard Mater Adv 4:100018. doi:10.1016/j.hazadv.2021.100018

[66] Sakai M, Tomiyama Y, Mori JF, Kanaly RA. 2022. Growth of Sphingobium barthaii KK22 on 1-ethylnaphthalene reveals diverse oxidative transformations and a complex metabolite profile. Int Biodeterior Biodegrad 175:105500. doi:10.1016/j.ibiod.2022.105500

[67] Takeshita T, Kanaly RA. 2019. In vitro DNA/RNA adductomics to confirm DNA damage caused by benzo[a]pyrene in the Hep G2 Cell Line. Front Chem 7:491. doi:10.3389/fchem.2019.00491 31338364 PMC6629907

[68] Jumper J, Evans R, Pritzel A, Green T, Figurnov M, Ronneberger O, Tunyasuvunakool K, Bates R, Žídek A, Potapenko A, et al.. 2021. Highly accurate protein structure prediction with AlphaFold. Nature 596:583–589. doi:10.1038/s41586-021-03819-2 34265844 PMC8371605

[69] Dürr SL, Levy A, Rothlisberger U. 2023. Metal3D: a general deep learning framework for accurate metal ion location prediction in proteins. Nat Commun 14:2713. doi:10.1038/s41467-023-37870-6 37169763 PMC10175565

[70] Trott O, Olson AJ. 2010. AutoDock Vina: improving the speed and accuracy of docking with a new scoring function, efficient optimization, and multithreading. J Comput Chem 31:455–461. doi:10.1002/jcc.21334 19499576 PMC3041641

[71] Eberhardt J, Santos-Martins D, Tillack AF, Forli S. 2021. AutoDock Vina 1.2.0: new docking methods, expanded force field, and python bindings. J Chem Inf Model 61:3891–3898. doi:10.1021/acs.jcim.1c00203 34278794 PMC10683950

