# Supplementary material for: Identification of a putative novel polycyclic aromatic hydrocarbon-biodegrading gene cluster in a marine Roseobacteraceae bacterium Sagittula sp. MA-2
Source: Microbiol Spectr. 2024 Nov 27;13(1):e01074-24. doi: 10.1128/spectrum.01074-24 (PMC11705938; doi:10.1128/spectrum.01074-24)
Supplement: Figures S1 to S4 — Fig. S1: Gene map of the strain MA-2 plasmid p6. Fig. S2: Results of LC-HRMS of strain MA-2 extract. Fig. S3: Results of HCD of phenanthrene biotransformation products in strain MA-2. Fig. S4: Predicted 3D structure of the selected enzymes in strain MA-2. [file spectrum.01074-24-s0001.docx]

**Supplementary Information**

**Identification of a putative novel polycyclic aromatic hydrocarbon-biodegrading gene cluster in a marine *Roseobacteraceae* bacterium *Sagittula* sp. MA-2**

Mayuko Abe, Miharu Sakai, Robert A. Kanaly, and Jiro F. Mori

Graduate School of Nanobioscience, Yokohama City University, Japan

**Figure S1.** Gene map of the strain MA-2 plasmid p6 with the location of the putative PAH-degrading gene cluster. Rings from outside to the center represent predicted genes on the forward and reverse strand (colored by COG classifications as listed), GC content (black), and GC skew (yellow and purple).

**Figure S2.** Results of LC-HRMS analyses of strain MA-2 extract (above) and an authentic standard of 1,2-dihydroxynaphthalene (below) within the *m/z* range of 159.0400-159.0500.

**Figure S3.** Results of HCD of phenanthrene biotransformation products in strain MA-2. (A) Product II, [M – H]^-^ = 187, identified as 1-hydroxy-2-naphthoic acid. (B) Product VI, [M – H]^-^ = 219, identified as 2-carboxybenzalpyruvic acid.

**Figure S4.** Predicted three-dimensional structure of the selected enzymes in strain MA-2 and results of substrate docking simulations. (**A**) Predicted structure of the large subunit of ARHD and docking simulation result for phenanthrene (magenta). (**B**) Predicted structure of ARCD and docking simulation result for 3,4-dihydroxyphenanthrene (pink). (**C**) Predicted structure of GDO and docking simulation results for gentisic acid (green) and 1H2N (yellow).

**Figure S1.** Gene map of the strain MA-2 plasmid p6 with the location of the putative PAH-degrading gene cluster. Rings from outside to the center represent predicted genes on the forward and reverse strand (colored by COG classifications as listed), GC content (black), and GC skew (yellow and purple).

**
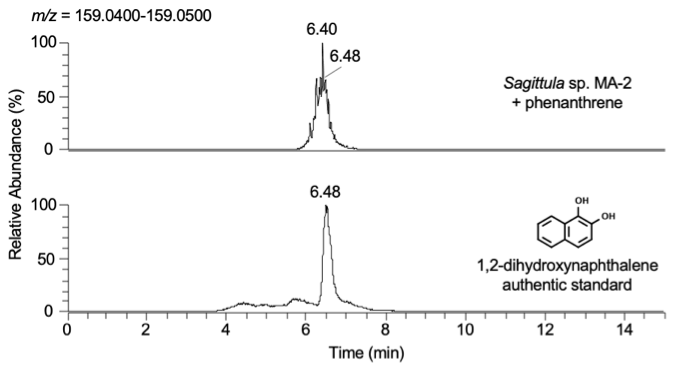
**

**Figure S2.** Results of LC-HRMS analyses of strain MA-2 extract (above) and an authentic standard of 1,2-dihydroxynaphthalene (below) within the *m/z* range of 159.0400-159.0500.

**Figure S3.** Results of HCD of phenanthrene biotransformation products in strain MA-2. (A) Product II, [M – H]^-^ = 187, identified as 1-hydroxy-2-naphthoic acid. (B) Product VI, [M – H]^-^ = 219, identified as 2-carboxybenzalpyruvic acid.

**Figure S4.** Predicted three-dimensional structure of the selected enzymes in strain MA-2 and results of substrate docking simulations. (**A**) Predicted structure of the large subunit of ARHD and docking simulation result for phenanthrene (magenta). (**B**) Predicted structure of ARCD and docking simulation result for 3,4-dihydroxyphenanthrene (pink). (**C**) Predicted structure of GDO and docking simulation results for gentisic acid (green) and 1H2N (yellow).
